# Accurate and cost-effective generation of a genetic risk score direct from blood lysates

**DOI:** 10.1186/s12967-024-05949-3

**Published:** 2024-12-20

**Authors:** Jonathan M. Locke, Benjamin Spurrier, Thomas W. Laver, Jayne A.L. Houghton, Kevin Colclough, Michael N. Weedon, Richard A. Oram

**Affiliations:** 1 Department of Clinical and Biomedical Sciences, Faculty of Health and Life Sciences, University of Exeter, Level 3, RILD Building, Barrack Road, Exeter, EX2 5DW UK; 2 Exeter Genomics Laboratory, Royal Devon University Healthcare NHS Foundation Trust, Exeter, UK

Dear editor,

Genetic risk scores (GRS), also known as polygenic risk scores, have utility in disease prediction, diagnosis, prognosis, clinical management, and treatment [1]. GRS are calculated by summing the effects of individual disease risk alleles, with single nucleotide polymorphisms (SNPs) most often assayed. SNP genotyping protocols typically require DNA to be purified, quantified, diluted, and stored. The complete costs (including reagents, equipment, staff, and energy use) of these pre-genotyping steps can be considerable and may impede clinical implementation.

Blood remains a common source of DNA for genetic testing, but its complex composition means SNP genotyping without purification of DNA is challenging. A variety of SNP genotyping methods (e.g., next-generation sequencing, microarray, real-time PCR) are available but there is a lack of user-ready protocols available for genotyping of user-defined SNPs without DNA purification. The TaqMan Sample-to-SNP kit (Thermo Fisher Scientific, Waltham, USA) described in a publication in 2012 [2] is an exception, but this method has seemingly not been widely adopted; Web of Science™ states this publication as having been cited only five times. As attention turns to clinical implementation of GRS, we considered that the potential cost-effectiveness of the TaqMan Sample-to-SNP method meant it deserved further appraisal. Here we present our findings on using the TaqMan Sample-to-SNP method to determine a 10-SNP type 1 diabetes genetic risk score (T1D-GRS) with utility in diabetes classification [3].

For 259 individuals referred for monogenic diabetes testing at Exeter Genomics Laboratory (UK), we froze the whole blood (collected by venepuncture into EDTA-coated tubes) that remained after routine DNA extraction. Given the minor allele frequencies of the SNPs, we predicted that we would detect 29/30 possible genotypes. We thawed 20 samples at a time and followed the TaqMan Sample-to-SNP kit protocol without preamplification for blood samples (available at https://assets.thermofisher.com/TFS-Assets/LSG/manuals/cms_057275.pdf). Using a Biomek i7 automated workstation (Beckman Coulter, Brea, USA) 384-well qPCR plates were set up. Each 5 µl reaction consisted of 2.5 µl TaqPath ProAmp Master Mix, 0.125 µl 40X Custom TaqMan SNP Genotyping Assay (assay IDs available upon request), 1.375 µl nuclease-free water and 1 µl of crude blood lysate. All qPCR plates contained DNA samples from 20 individuals and assayed all 10 SNPs. Plates were loaded into a QuantStudio 12 K Flex (Thermo Fisher Scientific) for thermocycling and fluorescence detection and genotypes called using the Connect Genotyping App (Thermo Fisher Scientific). Using purified DNA, and as previously described [4], the same genetic risk score was determined using Randox Biochip Array Technology (Randox, Crumlin, UK).

The TaqMan Sample-to-SNP protocol (*n* = 13 qPCR plates) resulted in an initial SNP call for 2556/2590 (98.7%) reactions and a complete GRS (all 10 SNPs called) for 237/259 (91.5%) samples. Repeating all samples with an incomplete GRS once (*n* = 22) resulted in a complete GRS for 256 (98.8%) samples. GRS concordance with Randox Biochip was very high (r^2^ = 0.99, *p* < 0.0001; Fig. 1) with 250/256 (97.7%) samples having an identical GRS. Using sequencing to resolve the remaining discordant SNP calls led us to estimate that 2/2578 (0.08%) TaqMan Sample-to-SNP genotypes were erroneous, an error rate that compares favourably with genotyping methods using purified DNA [4]. A subset of the blood samples (139/259) were also sent to Randox Laboratories for genotyping using a multiplex PCR protocol, with blood as input, followed by detection using Randox’s Biochip technology. 1384/1390 (99.6%) genotypes and 133/139 (95.7%) GRS were identical to those determined using the Biochip method with purified DNA as input (r^2^ = 0.98, *p* < 0.0001; Supp. Figure 1).

The per-sample reagent costs for cell lysis and DNA stabilisation using the TaqMan Sample-to-SNP protocol are £0.41–0.65 (depending on product size). This compares to reagent costs for commonly used extraction kits that purify DNA of £2.00-£3.20 (Table 1). So, whilst there are SNP genotyping assays cheaper than TaqMan available [5], we estimate the reagent costs of genotyping ~ 10 SNPs using TaqMan assays and blood lysates is no greater than using any of the cheaper genotyping methods that require pure DNA. This calculation does not consider benefits of the crude extraction method such as no requirement for equipment use (e.g., centrifuges, heat blocks) and its rapidity (~ 5 min) which are likely to result in significant cost savings with respect to technician time and resource use.

**Fig. 1:**
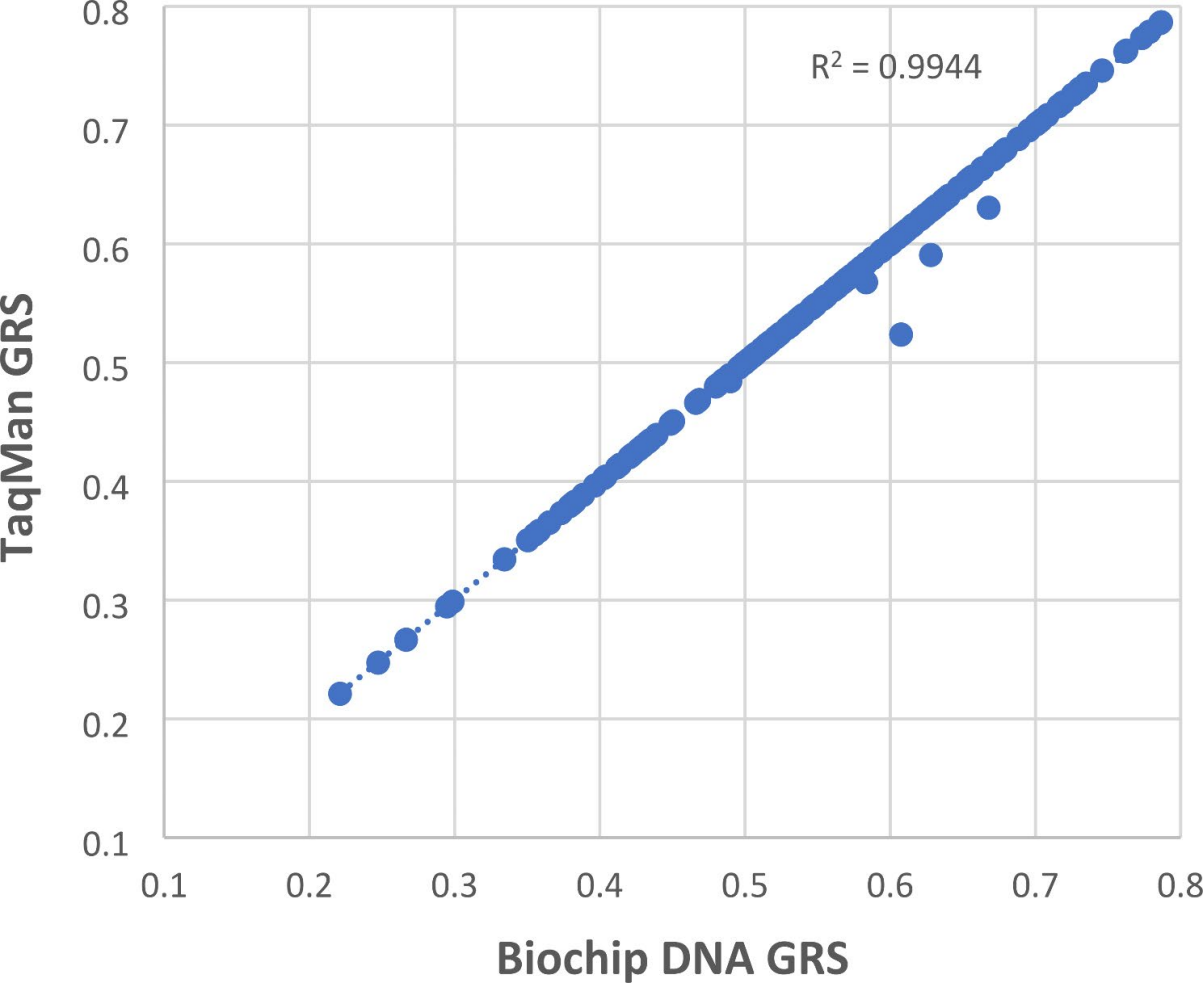
Correlation between the 10-SNP T1D GRS determined using Randox Biochip (with purified DNA as input) and TaqMan Sample-to-SNP (with crude venous blood lysate as input) in 256 individuals

**Table 1:** Cost of the crude lysate kit (DNA Extract All Reagents Kit) that is part of the TaqMan Sample-to-SNP kit protocol compared to costs of kits associated with extraction and purification of DNA that are available from the same vendor (Thermo Fisher Scientific). Prices are those stated on the Thermo Fisher Scientific website on 1st November 2024 and taking into account price of kit and number of samples that can be processed. Range of prices was calculated based on largest and smallest product sizes available

Consumable	Isolation technology	Company	Price per sample/10-SNP GRS
DNA Extract All Reagents Kit	Crude	Applied Biosystems™	£0.41-£0.65
PureLink™ Genomic DNA Mini Kit	Silica Spin column	Invitrogen™	£2.00-£2.80
GeneJET Whole Blood Genomic DNA Purification Mini Kit	Spin column	Thermo Fisher Scientific™	£2.55-£2.80
MagMAX™ DNA Multi-Sample Kit	Magnetic bead	Invitrogen™	£3.20

We have shown that a 10-SNP GRS can be accurately and cost-effectively determined using TaqMan chemistry and blood lysates. We report that genotyping accuracy is not significantly affected by having a relatively small number of different DNA samples on each plate (*n* = 20 excluding positive controls). The ability to determine GRS accurately and rapidly in small batches of samples is important as this reflects a typical clinical diagnostics situation. In summary, we urge investigators to consider whether DNA purification is necessary for calculation of all GRS, particularly when only a small number of SNPs need to be genotyped, and purified DNA is not required for further genetic investigations.

## Electronic supplementary material

Below is the link to the electronic supplementary material.

**Supplementary Material 1**: **Supplementary Fig. 1**: Correlation between the 10-SNP T1D GRS determined using Randox Biochip, either with purified DNA as input or direct from blood, in 139 individuals

## Data Availability

The datasets used and/or analysed during the current study are available from the corresponding author on reasonable request.
