# Supplementary material for: Accurate and cost-effective generation of a genetic risk score direct from blood lysates
Source: J Transl Med. 2024 Dec 20;22:1128. doi: 10.1186/s12967-024-05949-3 (PMC11662555; doi:10.1186/s12967-024-05949-3)
Supplement: Supplementary file 1 — Supplementary Material 1: Supplementary Fig. 1: Correlation between the 10-SNP T1D GRS determined using Randox Biochip, either with purified DNA as input or direct from blood, in 139 individuals [file 12967_2024_5949_MOESM1_ESM.docx]

**Supplementary Figure 1:** Correlation between the 10-SNP T1D GRS determined using Randox Biochip, either with purified DNA as input or direct from blood, in 139 individuals.
